# Editorial: Current Advances in Affective Neuroscience

**DOI:** 10.3389/fnins.2020.00338

**Published:** 2020-04-09

**Authors:** Benjamin Becker, Jiaojian Wang, Maria Bobes, Keith M. Kendrick

**Affiliations:** ^1^MOE Key Laboratory for Neuroinformation, The Clinical Hospital of the Chengdu Brain Science Institute, University of Electronic Science and Technology of China, Chengdu, China; ^2^Department of Cognitive Neuroscience, Cuban Neuroscience Center, Havana, Cuba

**Keywords:** affective neurocircuitry, affective neuroscience, depression, oxytocin, serotonin

Affective neuroscience is a rapidly progressing field and researchers are developing and employing innovative strategies to understand the neural circuitry and transmitter systems involved in affective processes and how they become dysfunctional in mental disorders. Recent epidemiological surveys have established disorders characterized by affective dysfunctions as one of the leading causes of disabilities worldwide and it is therefore of great importance to gain better insights into the key neural and molecular mechanisms underlying processing of affective stimuli and responses, how these can become dysfunctional and how they can be targeted by drug or other therapies. Our Frontiers Research Topic *Current Advances in Affective Neuroscience* aimed at providing an overview on the current developments and methodological advances in the field, encompassing animal models, and preclinical as well as clinical research in humans. The articles that were published in the Research Topic emphasize the breath-taking methodological advances in the field and the increasing aim of the community to contribute to the development of brain-based markers and novel treatments for disorders characterized by emotional dysregulations, as well as to bridge the gap between laboratory experiments and real life.

A number of authors emphasized the integration of novel methodological approaches rooted in computational sciences to facilitate progress in affective neuroscience. As such Jiang et al. proposed and evaluated a novel computational framework that employs a cortical folding pattern-guided approach to further delineate the emotional brain networks in the human brain, Markett et al. provided an overview and framework for the integration and application of recent developments in network neuroscience to describe emotional brain processes on the network level, and, Shani et al., proposed a machine learning based approach to optimize the efficacy of cognitive trainings, thus bridging the gap between methodological developments and clinical application. The increasing interest in the field to apply affective neuroscience inspired approaches to clinical questions, particularly novel treatment development, is further underscored by a number of preclinical studies. Employing sophisticated methodological strategies in rodent models Yuan et al. emphasized the importance of social company as a resource to attenuate fear renewal, Chen et al. demonstrated that the anxiolytic effects of Gan-Mai-Da-Zao, a traditional Chinese medicine, may be mediated by GABAergic and serotonergic receptors, while Rafa-Zablocka et al. reported evidence for the pivotal role of CREB in serotonergic neurons in the regulation of BDNF which may maintain the anti-depressive effects of Fluoxetine, a finding that resonates with the report from Han et al. that a BDNF-related imbalance of Copine 6 expression and synaptic plasticity may play a crucial role in the development of stress-associated depressive behavior. A review by Davis and Montag bridged the gap between animal models and human research by providing a cross-species perspective on Jaak Panksepp's affective neuroscience approach.

The submissions involving human subjects spanned a broad methodological range further emphasizing the rapid growth and extension of the field, both in terms of basic and clinical research. Submissions included behavioral genetic and psychophysiological approaches, such that Lischke, Weippert et al. demonstrated that inter-individual differences in heart-rate variability associate with emotion regulation capacities and that specifically individual variations in genotype-dependent catecholamine metabolism may account for facial emotion recognition performance (Lischke, Pahnke et al.), while other studies employed fMRI alone to determine the neural basis of positive and negative social interactions (Gao et al.) or in combination with pharmacological challenge strategies to demonstrate that the role of the neuropeptide oxytocin in emotional empathy is mediated by the amygdala and generalizes across cultures (Geng et al.) and that it modulates cognitive appraisal of close intimate relationships (Aguilar-Raab et al.), whereas a tryptophan depletion genetic imaging study by Klasen et al. emphasized the role of serotonergic amygdala-frontal connections in aggression. The increasing translation of affective neuroscience approaches to emotional brain disorders was reflected in the submission of studies employing fMRI to determine the importance of altered insular intrinsic networks in autism spectrum disorder (Xu et al.), deficient functional interactions between the ventral striatum and paralimbic regions (Bai et al.) as well as global functional communication deficits (Zhang et al.) in depression, while intrinsic connectivity measures may also represent a treatment sensitive marker for Electroconvulsive Therapy in this disorder (Wei et al.). Finally, two studies aimed at bridging the gap between experiments in the laboratory and the real-world, with a review by Leibold and Schruers stressing the importance of real-life assessments and epigenetic approaches in panic disorders and Pahnke et al. suggesting that oral contraceptives may impair complex emotion recognition in women.

In conclusion the Research Topic reflects that Affective Neuroscience increasingly integrates different methodological developments to delineate the neural foundations of emotional processing across species and aims at translating these findings in the context of determining brain-based markers for mental disorders and developing novel treatments for these disorders.

## Author Contributions

BB and KK drafted the manuscript. JW and MB critically revised the manuscript.

### Conflict of Interest

The authors declare that the research was conducted in the absence of any commercial or financial relationships that could be construed as a potential conflict of interest.

